# Optimizing sheep B-cell epitopes in *Echinococcus granulosus* recombinant antigen P29 for vaccine development

**DOI:** 10.3389/fimmu.2024.1451538

**Published:** 2024-08-14

**Authors:** Jihui Yang, Yongxue Lv, Yazhou Zhu, Jiahui Song, Mingxing Zhu, Changyou Wu, Yong Fu, Wei Zhao, Yinqi Zhao

**Affiliations:** ^1^ Center of Scientific Technology, Ningxia Medical University, Yinchuan, China; ^2^ Ningxia Key Laboratory of Prevention and Treatment of Common Infectious Diseases, Ningxia Medical University, Yinchuan, China; ^3^ School of Basic Medicine, Ningxia Medical University, Yinchuan, China; ^4^ Institute of Immunology, Zhongshan School of Medicine, Sun Yat-sen University, Guangzhou, China; ^5^ Qinghai Academy of Animal Sciences and Veterinary Medicine, Qinghai University, Xining, China

**Keywords:** *Echinococcus granulosus*, sheep, recombinant antigen P29, B cell epitopes, vaccine

## Abstract

**Background:**

*Echinococcus granulosus* is a widespread zoonotic parasitic disease, significantly impacting human health and livestock development; however, no vaccine is currently available for humans. Our preliminary studies indicate that recombinant antigen P29 (rEg.P29) is a promising candidate for vaccine.

**Methods:**

Sheep were immunized with rEg.P29, and venous blood was collected at various time points. Serum was isolated, and the presence of specific antibodies was detected using ELISA. We designed and synthesized a total of 45 B cell monopeptides covering rEg.P29 using the overlap method. ELISA was employed to assess the serum antibodies of the immunized sheep for recognition of these overlapping peptides, leading to the preliminary identification of B cell epitopes. Utilizing these identified epitopes, new single peptides were designed, synthesized, and used to optimize and confirm B-cell epitopes.

**Results:**

rEg.P29 effectively induces a sustained antibody response in sheep, particularly characterized by high and stable levels of IgG. Eight B-cell epitopes of were identified, which were mainly distributed in three regions of rEg.P29. Finally, three B cell epitopes were identified and optimized: rEg.P29_71-90_, rEg.P29_151-175_, and rEg.P29_211-235_. These optimized epitopes were well recognized by antibodies in sheep and mice, and the efficacy of these three epitopes significantly increased when they were linked in tandem.

**Conclusion:**

Three B-cell epitopes were identified and optimized, and the efficacy of these epitopes was significantly enhanced by tandem connection, which indicated the feasibility of tandem peptide vaccine research. This laid a solid foundation for the development of epitope peptide vaccine for *Echinococcus granulosus*.

## 1 Introduction

*Echinococcus granulosus* is a zoonotic parasitic disease caused by the larvae of the *Echinococcus* tapeworm, which parasitizes animals, including humans. It is globally distributed and prevalent in regions like Eastern Europe, East Africa, the Middle East, and Central Asia, particularly in areas with advanced animal husbandry (1, 2). This disease not only poses a severe threat to human health but also adversely affects the development of animal husbandry, leading to substantial medical and economic losses (3–5). Vaccines are a crucial and effective method for the prevention and control of epidemics, offering benefits such as high safety, no residue, and no withdrawal period for animals (6). The main vaccine types researched for *Echinococcus granulosus* include traditional, genetically engineered, nucleic acid, and peptide vaccines. Peptide vaccines are immunogenic vaccines designed and synthesized based on the amino acid sequence of an epitope from a known or predicted effective protective antigen (7, 8). Their simplicity in preparation, relatively stable structure, and absence of infection risk makes them a focal point in new vaccine research.

Screening and identifying dominant epitopes are essential for developing epitope-based vaccines. Optimizing antigen screening at the epitope level can induce a more effective immune response, ensuring immune specificity and safety (9, 10). Our group successfully cloned and constructed the recombinant antigen P29 (rEg.P29) earlier, which induced superior cellular and humoral immune responses in mice and sheep, providing 96.6% and 94.8% immune protection, respectively. These findings suggest that rEg.P29 is a promising candidate vaccine against *Echinococcus granulosus* (11, 12). We conducted rEg.P29 epitope peptide vaccine studies in mice, identifying T-cell and B-cell epitopes (13, 14), that elicited strong cellular and humoral immune responses in mice (15). However, data on peptide vaccines for sheep, the most suitable hosts for *Echinococcus granulosus*, are lacking. Developing and promoting the rEg.P29 peptide vaccine for sheep holds significant practical value for disease prevention and control.

In this study, we designed and synthesized single amino acid peptides covering rEg.P29 using the overlap method. We used enzyme linked immunosorbent assay (ELISA) to detect antibody recognition of overlapping peptides and initially screened B-cell epitopes. Following this, new peptides were designed and synthesized, with the B cell epitopes being finalized through further optimization and characterization.

## 2 Materials and methods

### 2.1 Preparation of antigen

rEg.P29 was prepared using a recombinant expression plasmid stored in our laboratory, following the specific protocol previously described (16). Briefly, sterile LB liquid medium was prepared, containing 0.1 mM isopropyl β-D-thiogalactoside (IPTG, Invitrogen, Waltham, USA). The preserved strain was inoculated into the LB liquid medium and incubated at 37°C for 10 h. rEg.P29 was then purified using a Ni-NTA His-Tag purification kit (Merck, Kenilworth, USA), and finally the protein was eluted and dissolved by Elution Buffer containing urea, and endotoxins were eliminated with an endotoxin removal kit (Genscript, Nanjing, China). The endotoxin-free purified rEg.P29 underwent protein concentration assessment using a BCA kit (KeyGen Biotech, Nanjing, China), and protein purity was confirmed by sodium dodecyl sulfate polyacrylamide gel electrophoresis (SDS-PAGE).

### 2.2 Animal immunization and sample collection

**Figure 1:**
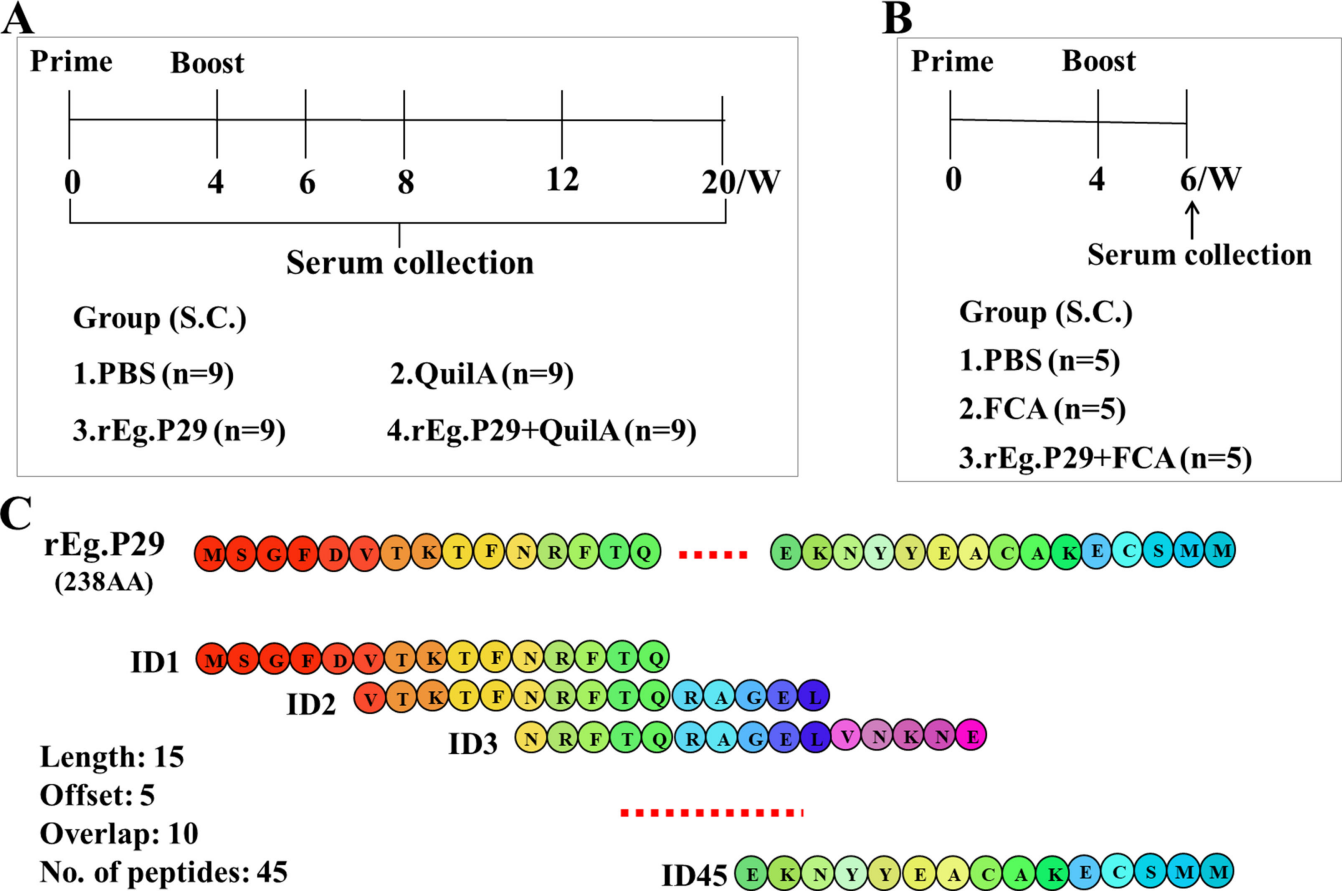
Animal immune grouping and epitope peptide design strategies. **(A)** Sheep (n=9/group) were immunized by subcutaneous injection in the abdomen, 4 weeks between initial and booster vaccination. Peripheral blood was collected at the designated times. **(B)** Mice were immunized subcutaneously via the abdomen, with an interval of 4 weeks between the initial and booster immunizations, and serum was collected two weeks after the booster immunization. **(C)** The 45 overlapping single peptides were designed with a length of 15 amino acids, a step of 5 amino acids and an overlap of 10 amino acids, covering 238 amino acids of rEg.P29.

Thirty-six female Chinese Yan chi Tan sheep, aged 4-6 months, were randomly divided into four groups. Each group received subcutaneous immunizations with primary and booster doses at 4-week intervals: PBS group (1 mL PBS), Quil A adjuvant group (1 mg Quil A, InvivoGen, San Diego, USA), rEg.P29 group (50 μg rEg.P29), and rEg.P29+Quil A group (50 μg rEg.P29 with 1 mg Quil A). Sheep peripheral blood was collected via the jugular vein at different time points, and serum was isolated. Fifteen 6–8-week-old female C57BL/6 mice were randomly divided into three groups: PBS group (1 mL PBS), Freund’s adjuvant group (Freund’s adjuvant), and rEg.P29+Freund’s adjuvant group (20 μg rEg.P29 with Freund’s adjuvant). Mice received booster immunizations two weeks after the initial dose, using Freund’s complete and incomplete adjuvants (Sigma-Aldrich, St. Louis, USA). Mice were anesthetized with sodium pentobarbital via tail vein injection for serum collection. Details of animal immunization and sample collection are illustrated in **Figures 1A, B**.

### 2.3 Epitope peptide design and synthesis

Following the overlapping peptides design principle, each designed single peptide spanned 15 amino acids, with a step size of 5 amino acids and an overlap of 10 amino acids. We designed a total of 45 overlapping single peptides to encode the 238 amino acids of rEg.P29 (**Figure 1C**; **Table 1**). To aid in the screening of single peptides, three adjacent single peptides were mixed in equal proportions, resulting in 15 mixed peptide groups and one comprehensive mix of all single peptides. Sangon Biotech (Shanghai, China) synthesized the designed peptides with a purity of ≥ 98%, ensuring they were sterile and endotoxin-free. According to the buffer recommended in the peptide synthesis report, the peptides were dissolved in a 1 mg/mL solution and stored at -80°C for use.

**Table 1:** Designed overlapping peptides information in the study.

Peptide No.	Amino acid positions	Sequences	Length	Hydrophilic residue ratio	Basic/acidic
ID1	rEg.P29_1-15_	MSGFDVTKTFNRFTQ	15	40%	neutral
ID2	rEg.P29_6-20_	VTKTFNRFTQRAGEL	15	40%	basic
ID3	rEg.P29_11-25_	NRFTQRAGELVNKNE	15	60%	neutral
ID4	rEg.P29_16-30_	RAGELVNKNEKTSYP	15	53%	neutral
ID5	rEg.P29_21-35_	VNKNEKTSYPTRTSD	15	60%	neutral
ID6	rEg.P29_26-40_	KTSYPTRTSDLIHEI	15	40%	neutral
ID7	rEg.P29_31-45_	TRTSDLIHEIDQMKA	15	47%	neutral
ID8	rEg.P29_36-50_	LIHEIDQMKAWISKI	15	40%	neutral
ID9	rEg.P29_41-55_	DQMKAWISKIITATE	15	40%	neutral
ID10	rEg.P29_46-60_	WISKIITATEEFVDI	15	33%	acidic
ID11	rEg.P29_51-65_	ITATEEFVDINIASK	15	40%	acidic
ID12	rEg.P29_56-70_	EFVDINIASKVADAF	15	40%	acidic
ID13	rEg.P29_61-75_	NIASKVADAFQKNKE	15	60%	neutral
ID14	rEg.P29_66-80_	VADAFQKNKEKITTT	15	60%	neutral
ID15	rEg.P29_71-85_	QKNKEKITTTDKLGT	15	53%	basic
ID16	rEg.P29_76-90_	KITTTDKLGTALEQV	15	33%	neutral
ID17	rEg.P29_81-95_	DKLGTALEQVASQSE	15	53%	acidic
ID18	rEg.P29_86-100_	ALEQVASQSEKAAPQ	15	53%	acidic
ID19	rEg.P29_91-105_	ASQSEKAAPQLSKML	15	53%	neutral
ID20	rEg.P29_96-110_	KAAPQLSKMLTEASD	15	53%	neutral
ID21	rEg.P29_101-115_	LSKMLTEASDVHQRM	15	47%	neutral
ID22	rEg.P29_106-120_	TEASDVHQRMATARK	15	47%	basic
ID23	rEg.P29_111-125_	VHQRMATARKNFNSE	15	53%	basic
ID24	rEg.P29_116-130_	ATARKNFNSEVNTTF	15	47%	neutral
ID25	rEg.P29_121-135_	NFNSEVNTTFIEDLK	15	53%	acidic
ID26	rEg.P29_126-140_	VNTTFIEDLKNFLNT	15	40%	acidic
ID27	rEg.P29_131-145_	IEDLKNFLNTTLSEA	15	47%	acidic
ID28	rEg.P29_136-150_	NFLNTTLSEAQKAKT	15	47%	neutral
ID29	rEg.P29_141-155_	TLSEAQKAKTKLEEV	15	53%	neutral
ID30	rEg.P29_146-160_	QKAKTKLEEVRLDLD	15	60%	neutral
ID31	rEg.P29_151-165_	KLEEVRLDLDSDKTK	15	67%	acidic
ID32	rEg.P29_156-170_	RLDLDSDKTKLKNAK	15	67%	basic
ID33	rEg.P29_161-175_	SDKTKLKNAKTAEQK	15	67%	basic
ID34	rEg.P29_166-180_	LKNAKTAEQKAKWEA	15	53%	basic
ID35	rEg.P29_171-185_	TAEQKAKWEAEVRKD	15	60%	neutral
ID36	rEg.P29_176-190_	AKWEAEVRKDESDFD	15	67%	acidic
ID37	rEg.P29_181-195_	EVRKDESDFDRVHQE	15	73%	acidic
ID38	rEg.P29_186-200_	ESDFDRVHQESLTIF	15	53%	acidic
ID39	rEg.P29_191-205_	RVHQESLTIFEKTCK	15	47%	basic
ID40	rEg.P29_196-210_	SLTIFEKTCKEFDGL	15	40%	acidic
ID41	rEg.P29_201-215_	EKTCKEFDGLSVQLL	15	47%	acidic
ID42	rEg.P29_206-220_	EFDGLSVQLLDLIRA	15	40%	acidic
ID43	rEg.P29_211-225_	SVQLLDLIRAEKNYY	15	47%	neutral
ID44	rEg.P29_216-230_	DLIRAEKNYYEACAK	15	47%	neutral
ID45	rEg.P29_221-235_	EKNYYEACAKECSMM	15	47%	acidic

Peptide information includes number, amino acid positions, sequences, length, hydrophilic residue ratio, and acid-base property.

### 2.4 Enzyme-linked immunosorbent assay

rEg.P29 (recognition antibody, positive control), along with single or mixed peptides, were diluted to 5 μg/mL by carbonate buffer (pH 9.6) and incubated overnight at 4°C in enzyme-labeled plates for encapsulation. The plates were then washed five times with PBST (PBS with 0.05% Tween-20) and subsequently blocked with 5% skim milk powder for 2 h at 37°C. After washing, diluted sheep or mouse serum, serving as the primary antibody, was added to the plate, and incubated for 1 hour at 37°C. Horseradish peroxidase (HRP)-conjugated anti-sheep immunoglobulin G (IgG), IgM, IgA, IgE, IgG1, IgG2 (ABD Serotec, Kidlington, UK), or anti-mouse IgG, IgM, IgA, IgE, IgG1, IgG2a, IgG2b, IgG2c, and IgG3 (Abcam, Cambridge, USA) were added and incubated for another hour at 37°C. Following this, the plate was washed, and 3,3’,5,5’-Tetramethylbenzidine (TMB) was introduced. The reaction was terminated with 2 M H2SO4. Absorbance was measured at 450 nm using a Multiskan SkyHigh Microplate Spectrophotometer (Thermo Fisher Scientific, MA, USA).

### 2.5 B-cell epitopes screening

Single- and mixed-peptide screening was conducted using sheep serum samples exhibiting the highest IgG antibody levels. Plates were coated with either peptides or rEg.P29, and the serum acted as the primary antibody. B-cell mixed peptides were identified by assessing IgG antibodies’ recognition using the ELISA method, as previously described. Subsequently, the corresponding single peptides of these mixed peptides were screened to pinpoint the B-cell epitopes.

### 2.6 B-cell epitopes identification and optimization

Based on the locations and amino acid sequences of the initially identified epitopes, new peptides were methodically designed, optimized, and subsequently synthesized. ELISA plates were coated with these newly synthesized peptides alongside rEg.P29, employing the same ELISA procedure as described previously for the screening of B-cell epitopes. Ultimately, this process led to the identification and optimization of sheep B-cell epitopes.

### 2.7 Statistical analysis

Data analysis and graphing were conducted using the Statistical Package for the GraphPad Prism 8.0 graphing software (SPSS) version 22.0. Comparisons between two groups were executed using an unpaired t-test, while comparisons involving two or more groups employed one-way ANOVA. Data are presented as either mean or mean ± standard deviation (SD). *P* < 0.05 is considered statistically significant.

## 3 Results

### 3.1 rEg.P29 induces a sustained and strong antibody response in sheep

**Figure 2:**
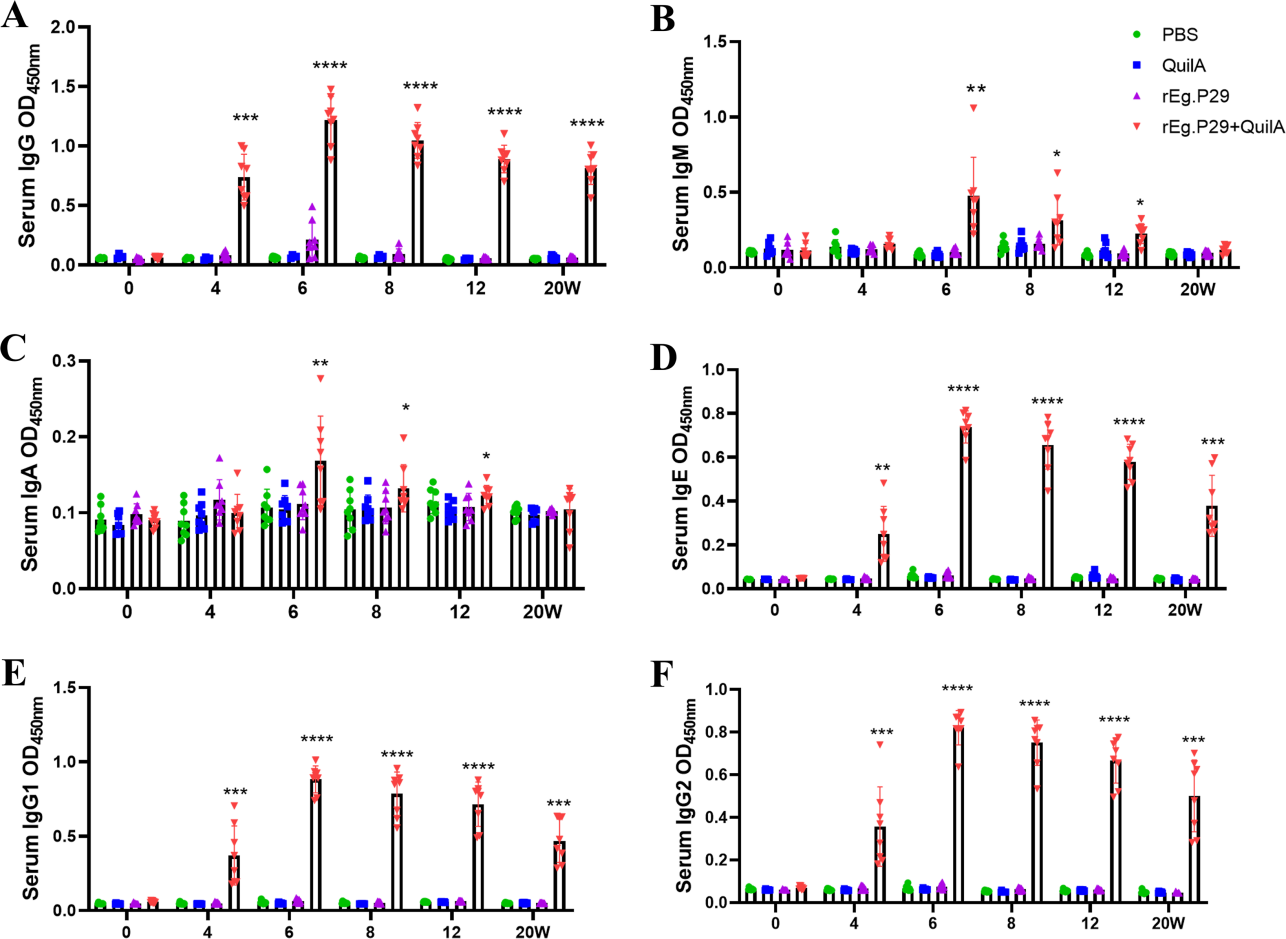
Serum antibody expression in rEg.P29-immunized sheep. **(A)** Expression of serum specific antibody IgG. **(B)** Expression of serum specific antibody IgM. **(C)** Expression of serum specific antibody IgA. **(D)** Expression of serum specific antibody IgE. **(E)** Expression of serum specific antibody IgG1. **(F)** Expression of serum specific antibody IgG2. 0 weeks represents primary immunization. Results presented as mean ± SD (*P < 0.05; **P < 0.01; ***P < 0.001; ****P < 0.0001).

Analysis of serum antigen-specific antibodies in sheep at various time points post-immunization revealed that immunization with rEg.P29, particularly when supplemented with the adjuvant QuilA, elicited high levels of specific IgG, IgM, IgE, IgG1, and IgG2 (**Figures 2A, B, D–F**). A modest amount of IgA was also detected (**Figure 2C**), with IgG showing the highest and most rapid increase. Notably, immunization with rEg.P29 alone also induced some level of IgG production (**Figure 2A**). All antibody types demonstrated a rapid increase following immunization, reaching a peak two weeks post-booster immunization. Over time, antibody levels gradually declined, with IgA and IgM decreasing more rapidly compared to a slower decline in IgG.

**Figure 3:**
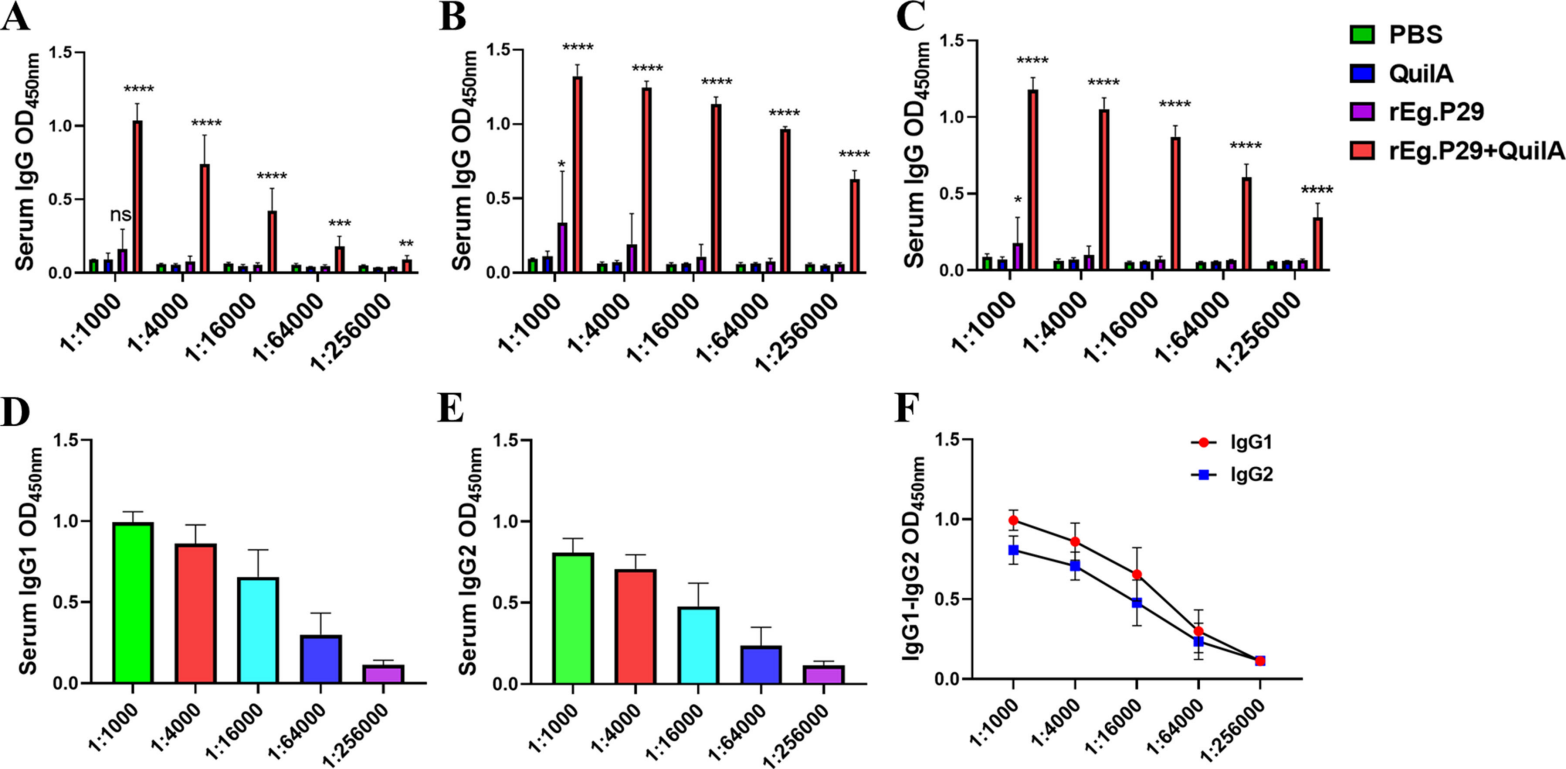
Serum IgG antibody titers determination in immunized sheep. **(A)** IgG antibody expression after doubling dilution of serum samples at week 4. **(B)** IgG antibody expression after doubling dilution of serum samples at week 6. **(C)** IgG antibody expression after doubling dilution of serum samples at week 8. **(D)** IgG1 antibody expression after doubling dilution of serum samples at week 6. **(E)** IgG2 antibody expression after doubling dilution of serum samples at week 6. **(F)** Comparison of IgG1 and IgG2 antibody expression after doubling dilution of serum samples at week 6. Results presented as mean ± SD (ns, *P* > 0.05; *P < 0.05; **P < 0.01; ***P < 0.001; ****P < 0.0001).

Sera collected at weeks 4, 6, and 8 were diluted from 1:1,000 to 1:256,000. Remarkably, even at a 256,000-fold dilution, high IgG titers were maintained (**Figures 3A–C**), particularly evident at week 6 (**Figure 3B**), which corresponds to two weeks post-booster immunization. At a 64,000-fold dilution of the week 6 serum, the levels of IgG subtypes IgG1 and IgG2 remained relatively high, with IgG1 levels surpassing those of IgG2 (**Figures 3D–F**). These findings strongly suggest that rEg.P29 effectively induces a sustained antibody response in sheep, particularly characterized by high and stable levels of IgG.

### 3.2 Preliminary screening of eight B-cell dominant epitopes

**Figure 4:**
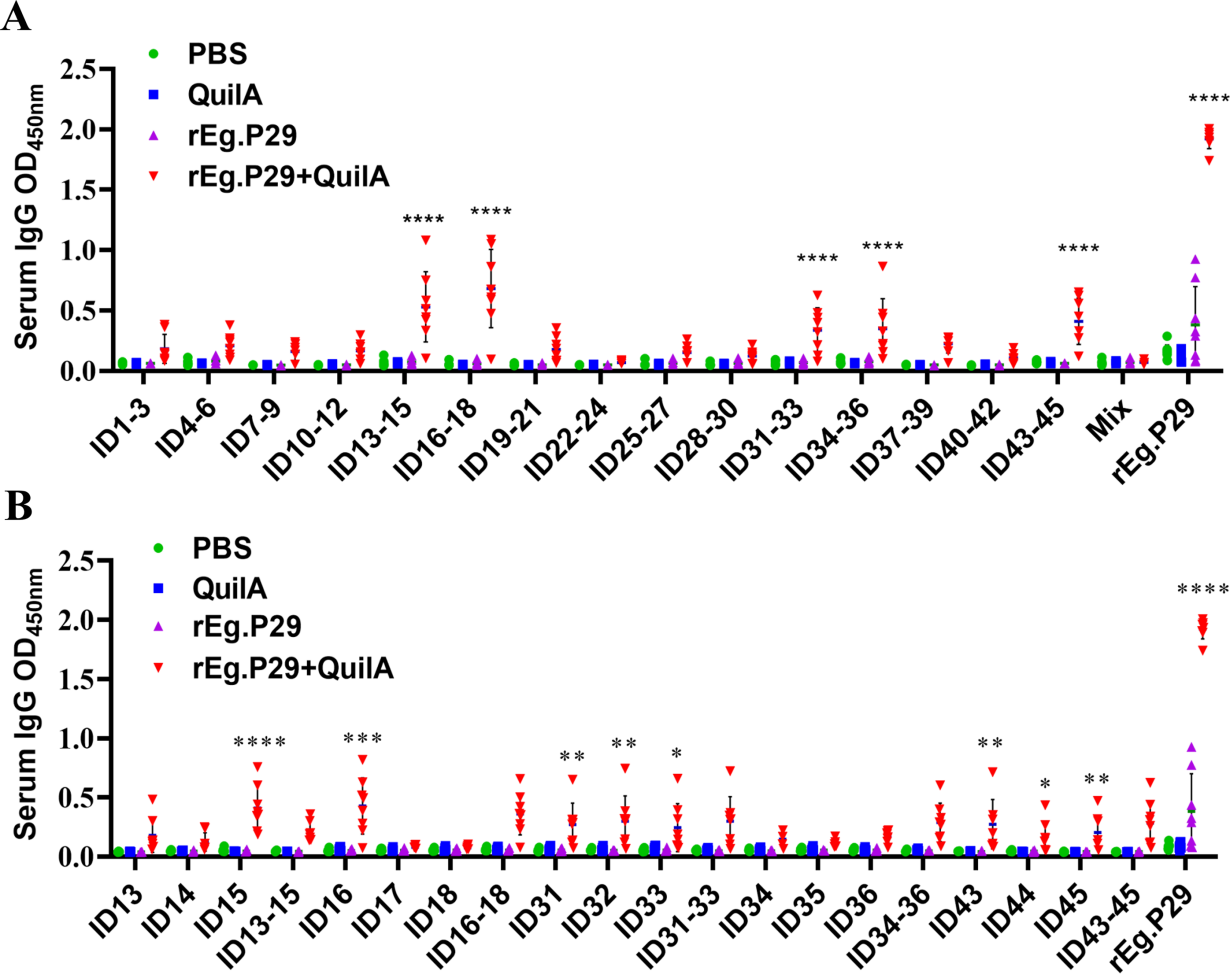
Preliminary screening of B-cell mixed peptides and single peptides. **(A)** Screening of B-cell mixed peptides by recognition of IgG antibodies. “Mix” means pools of 15 epitope peptides. **(B)** Screening of B-cell monopeptides by recognition with IgG antibodies. Results presented as mean ± SD (*P < 0.05; **P < 0.01; ***P < 0.001; ****P < 0.0001).

In this phase, fifteen pools of 3 epitope peptides (**Table 1**), each comprising three adjacent single peptides, were screened. Five pools of 3 epitope peptides were identified: ID13-15, ID16-18, ID31-33, ID34-36, and ID43-45 (**Figure 4A**). Notably, ID13-15 and ID16-18 elicited higher responses, significantly differing from the other pools of 3 epitope peptides. These five pools of 3 epitope peptides collectively encompass fifteen single peptides: ID13, ID14, ID15, ID16, ID17, ID18, ID31, ID32, ID33, ID34, ID35, ID36, ID43, ID44, and ID45. A subsequent screening was conducted on these 15 peptides.

Further analysis revealed that eight peptides were recognized in the immunoserum: ID15, ID16, ID31, ID32, ID33, ID43, ID44, ID45 (**Figure 4B**). These peptides correspond to the 71-85AA, 76-90AA, 151-165AA, 156-170AA, 161-175AA, 211-225AA, 216-230AA, and 221-235AA regions of rEg.P29, initially considered as potential linear B-cell epitopes. The identified epitopes were in three distinct regions of rEg.P29: ID15 and ID16 in the 71-90AA region, ID31, ID32, and ID33 in the 151-175AA region, and ID43, ID44, and ID45 in the 211-235AA region. The question arose whether these three regional peptides are more effective than the corresponding single peptides. To address this, further optimization, verification, and identification were undertaken.

### 3.3 Identification and optimization of three B-cell dominant epitopes

**Figure 5:**
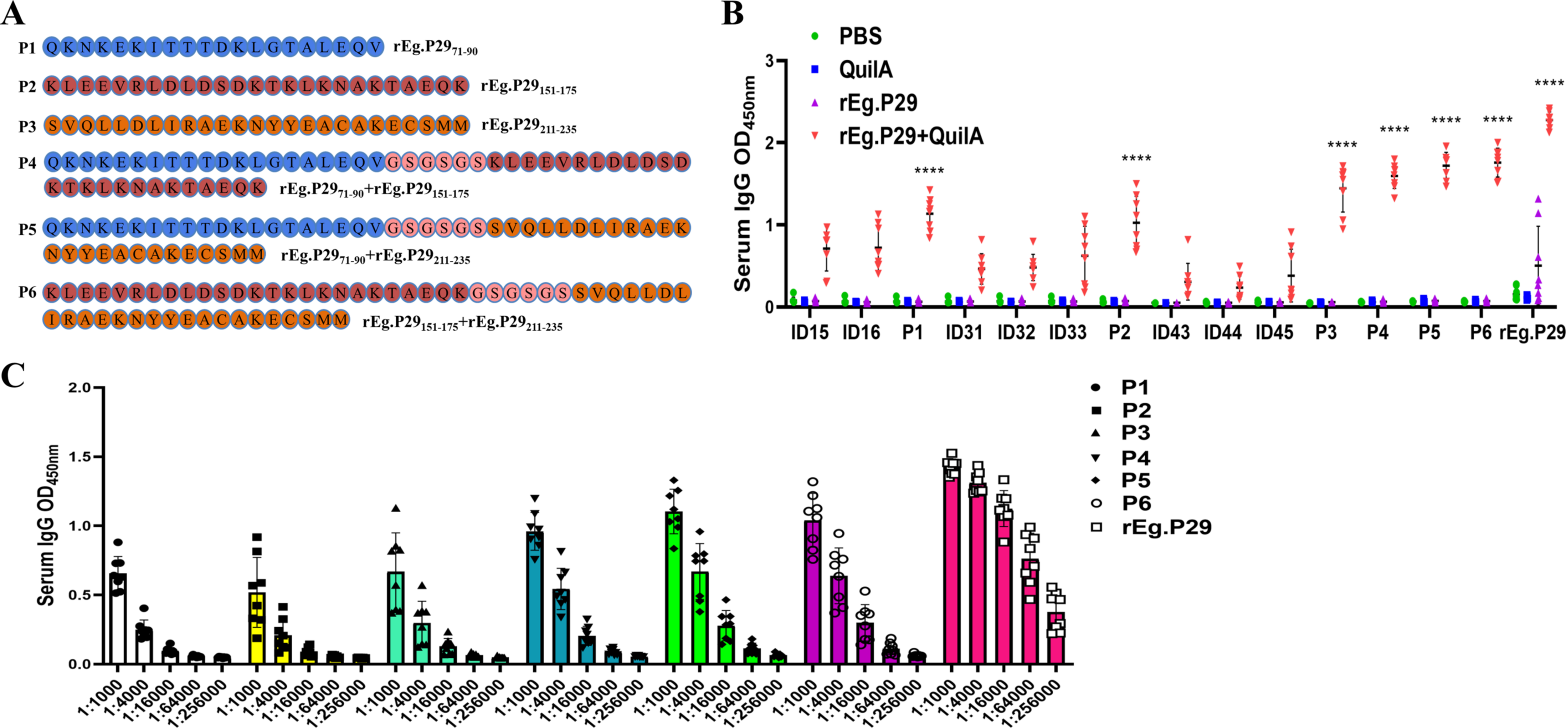
Optimized design strategies and identification of B-cell epitopes. **(A)** Three new single peptides were designed and connected in series two by two with GSGSGS in the middle. **(B)** Optimization and identification of B-cell epitopes by IgG antibody recognition (Statistical analysis was made within and between groups). **(C)** Doubling dilution determination of IgG antibodies to six B epitopes. Data from 9 sheep, results presented as mean ± SD (****P < 0.0001).

New single peptides corresponding to the regions 71-90AA, 151-175AA, and 211-235AA were synthesized. Additionally, three peptides were created by tandemly connecting two regions each, with GSGSGS tandem sequences inserted between them. This process resulted in six new single peptides (P1-P6), as depicted in **Figure 5A**. Antibody recognition tests revealed that P1 (71-90AA), P2 (151-175AA), and P3 (211-235AA) demonstrated markedly enhanced recognition compared to their respective individual peptides within each region. Notably, P3 exhibited superior efficacy (**Figures 5B, C**). The number of amino acids increased when P1, P2, and P3 were linked in tandem, enhancing their recognition beyond the pre-tandem levels. However, no significant difference in recognition was observed among the three tandem peptides (P4, P5, and P6), as shown in **Figure 5B**. Consequently, epitope peptides P1, P2, and P3 were identified as the three principal B-cell epitopes. It was also observed that the efficacy of these three epitopes significantly increased when they were linked in tandem.

### 3.4 Identified B-cell epitopes efficiently recognize antibodies in sheep and mice

**Figure 6:**
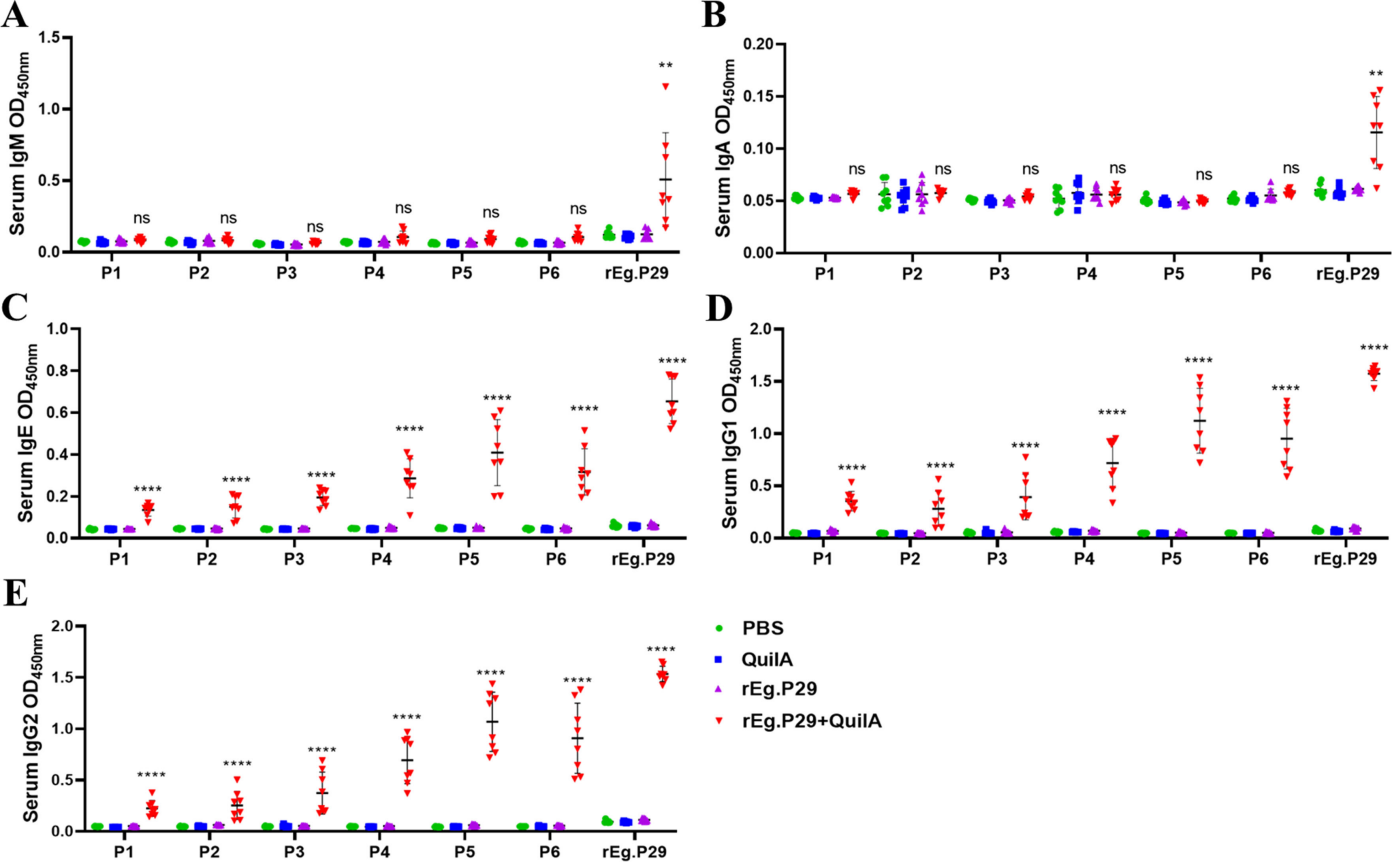
Recognition of sheep antibodies by optimized B cell epitopes. **(A)** Recognition of lgM by B cell epitopes. **(B)** Recognition of lgA by B cell epitopes. **(C)** Recognition of lgE by B cell epitopes. **(D)** Recognition of lgG1 by B cell epitopes. **(E)** Recognition of lgG2 by B cell epitopes. Results presented as mean ± SD (ns, *P* > 0.05; **P < 0.01; ****P < 0.0001).

The recognition of six single peptides, P1-P6, by IgM, IgA, IgE, IgG1, and IgG2 antibodies in sheep serum was observed. The results indicated that these peptides could not recognize IgM and IgA antibodies (**Figures 6A, B**), but they effectively recognized IgE, IgG1, and IgG2 antibodies. Notably, peptides P4, P5, and P6 showed superior recognition effects compared to P1, P2, and P3, with P5 demonstrating the most significant recognition impact on the three antibodies (**Figures 6C–E**).

**Figure 7:**
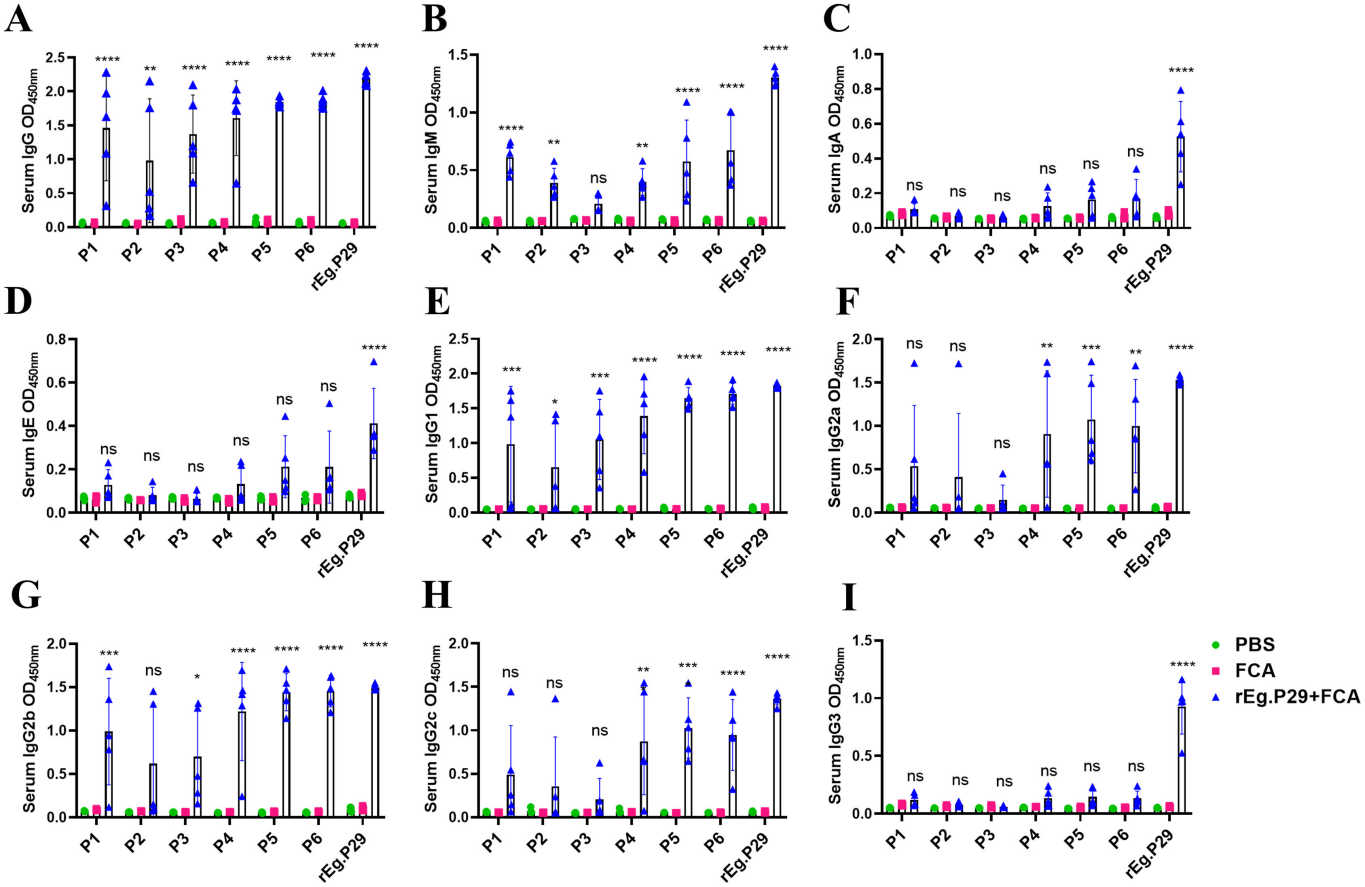
Recognition of mouse antibodies by optimized B cell epitopes. **(A)** Recognition of lgG by B cell epitopes. **(B)** Recognition of lgM by B cell epitopes. **(C)** Recognition of lgA by B cell epitopes. **(D)** Recognition of lgE by B cell epitopes. **(E)** Recognition of lgG1 by B cell epitopes. **(F)** Recognition of lgG2a by B cell epitopes. **(G)** Recognition of lgG2b by B cell epitopes. **(H)** Recognition of lgG2c by B cell epitopes. **(I)** Recognition of lgG3 by B cell epitopes. Results presented as mean ± SD (ns, *P* > 0.05; *P < 0.05; **P < 0.01; ***P < 0.001; ****P < 0.0001).

Similarly, the interaction of these six single peptides, P1-P6, with various antibodies in mouse serum was examined. The findings revealed that they could recognize IgG, IgM, IgG1, and IgG2b antibodies (**Figures 7A, B, E, G**), but failed to recognize IgA, IgE, and IgG3 antibodies (**Figures 7C, D, I**). Peptides P4, P5, and P6 exhibited enhanced recognition effects compared to P1, P2, and P3. Additionally, P4, P5, and P6 were able to recognize IgG2a and IgG2c antibodies, unlike P1, P2, and P3 (**Figures 7F, H**). These results demonstrate that the three B-cell epitopes, P4, P5, and P6, optimized through this study, can effectively recognize antibodies in both sheep and mice, qualifying them as dominant B-cell epitopes.

## 4 Discussion

Antigen-specific antibodies are crucial in antiparasitic infections and serve as a key index for evaluating humoral immunity (17, 18). Post-immunization of sheep with rEg.P29, the serum exhibited high titers of IgG, IgG1, and IgG2 antibodies, with levels rapidly increasing after booster immunization and remaining elevated until the 20th week. In earlier stages, our group employed rEg.P29 in conjunction with Freund’s complete/incomplete adjuvant for sheep immunization and infection (12), which is consistent with the current research results. Lalramhluna et al. infected two types of sheep with Haemonchus contortus and noted a significant increase in IgG1 and IgG2 levels in the serum of resistant sheep, indicating a more robust humoral immune response (19). Valizadeh et al. used the envelope antigen extracted from live Protocercaria for sheep immunization and observed a notable elevation in IgG antibody titer in the immunized group (20). Heath et al. established the correlation between IgG antibody levels and immune protection in organisms (21). The high and sustained levels of IgG, IgG1, and IgG2 antibodies induced by the vaccine are vital for resistance to parasitic infections (22, 23), which is corroborated by the antibody responses observed in this study. These findings suggest that rEg.P29 is effective in inducing humoral immune responses in sheep.

On the surface of an antigenic molecule, certain specific chemical groups determine the antigen’s specificity. These groups are known as antigenic determining groups or antigenic epitopes (24, 25), which act as functional units for antigen-receptor binding and play various roles in eliciting humoral and cellular immune responses (26, 27). In vaccine-induced protective immune responses, antigen-specific epitopes are predominantly involved, with the body’s immune response primarily targeting antigen-dominant epitopes (28, 29). Peptide vaccines, based on antigenic epitopes, are crucial in disease prevention (30, 31). Identifying antigenically dominant epitopes with protective effects is fundamental for developing epitope-based peptide vaccines, making the study of antigenic epitopes a critical methodology. The overlapping synthetic peptide method is commonly used for identifying cellular epitopes (32, 33), which is adopted in this study.

Peptide vaccines designed to effectively elicit humoral and/or cellular immune responses must incorporate epitopes capable of triggering the desired immune reaction. B-cell epitopes are typically categorized as either linear or conformational epitopes (34, 35). Due to the challenges in identifying conformational epitopes and the widespread use of linear epitopes, the latter have garnered more attention. In dogs, fine-grained *Echinococcus granulosus* tapeworm infections are chiefly mediated by antibody-specific B-cell antibodies (36), making protective B-cell epitope peptides critical for peptide vaccine development (37). Researchers have conducted extensive studies on echinococcosis peptide vaccines, focusing primarily on informatics analysis and the epitope peptides of the Eg95 gene. Woollard et al. synthesized four epitope peptides of Eg95, demonstrating their strong immunogenicity in inducing IgG1 and IgG2 antibodies in sheep. However, these peptides did not confer immune protection in sheep, indicating a need for further investigation into the mechanism of immune protection by epitope peptides (38). Esmaelizad et al. integrated five T-cell epitopes into a multicell epitope antigen, which induced mice achieved a protection rate of 99.6% (39). Currently, peptide vaccines for echinococcosis remain in the stages of informatics prediction and laboratory validation (40–42), with no mature peptide vaccines available yet for the prevention and treatment of echinococcosis. Our efforts are directed towards developing a multi-epitope peptide vaccine with effective immune-protective properties.

In this study, we screened and optimized B-cell epitopes by examining the interaction of overlapping peptides with specific IgG antibodies using ELISA. The dominant B-cell epitopes were identified as rEg.P29_71-90_ (P1), rEg.P29_151-175_ (P2), and rEg.P29_211-235_ (P3). It was confirmed that these dominant B-cell epitopes could recognize sheep-specific IgE, IgG1, and IgG2 antibodies, but they did not bind to specific IgM and IgA antibodies. This may be attributed to the relatively low levels of these two antibodies in the serum. When the three dominant epitopes were linked in tandem, their peptide recognition efficacy significantly exceeded that of the individual dominant epitopes. This improved recognition is likely due to the broader range of epitopes presented by a larger number of amino acids, suggesting the potential for developing multi-epitope peptide vaccines. Moreover, various peptides and peptides in tandem also identified mouse-specific IgG, IgM, IgG1, and IgG2b antibodies. Additionally, the tandem peptides were able to recognize mouse-specific IgG2a and IgG2c. This indicates that the B-cell epitopes screened and identified using sheep might also be effectively applicable in mice.

Screening of the few peptides identified did produce high OD values, but we must be concerned that the coating efficiency of peptides affects the results of peptide identification. This requires us to use certain methods to make the peptide encapsulation as homogeneous as possible, such as the use of labelling and antibody capture methods. Inhibition experiments can be performed to control the binding of rEg.P29 antibody bound to the enzyme labelled plate, allowing better control of the encapsulation efficiency. Antibodies that are highly reactive to peptides in ELISA are not necessarily neutralizing antibodies and may not be immunoprotective. At the same time, some of the peptides identified by the screen may be aggregated, resulting in obtaining peptides that may not be the results we desire. Through the mouse animal model, our research group screened and confirmed the dominant epitopes of T cells and B cells of rEg.P29. Immunizing mice with combined epitopes produced strong humoral and cellular immune effects, especially B cell epitopes (15). The protective effect of combined epitope vaccine on mouse infection model is being studied. Screening and identifying peptides with good immunoprotective effects is our goal. The binding of peptide-inducing antibodies to natural rEg.P29 would be a good indicator of potential efficacy. rEg.P29 is known to bind tightly to lipids, which may affect its ability to bind to peptide-inducing antibodies. This provides a better reference for our subsequent studies. Therefore, combined with the results of the mouse animal model, in the follow-up study, we will carry out the peptide vaccine protection effect study targeting B-cell epitopes.

## 5 Conclusion

Three dominant B-cell epitopes of rEg.P29 were successfully identified: rEg.P29_71-90_, rEg.P29_151-175_, and rEg.P29_211-235_. The efficacy of these epitopes was notably enhanced through tandem linkage, indicating the feasibility of conducting research on tandem peptide vaccines. This advancement lays a solid groundwork for the development of epitope-based peptide vaccine.

## Data availability statement

The original contributions presented in the study are included in the article/supplementary material. Further inquiries can be directed to the corresponding authors.

## Ethics statement

The animal study was approved by Ethics Committee of Ningxia Medical University. The study was conducted in accordance with the local legislation and institutional requirements.

## Author contributions

JY: Writing – original draft, Writing – review & editing. YL: Writing – original draft. YZZ: Writing – original draft. JS: Writing – original draft. MZ: Writing – review & editing. CW: Writing – review & editing. YF: Writing – original draft. WZ: Writing – review & editing. YQZ: Writing – review & editing.
